# Proliferative verrucous/multifocal leukoplakia: Updates and literature review “case report”

**DOI:** 10.1097/MD.0000000000033783

**Published:** 2023-05-26

**Authors:** Abdullah Alsoghier, Hamad AlBagieh, Lujain AlSahman, Rana Alshagroud, Roba AlSahman

**Affiliations:** a Oral Medicine and Diagnostic Sciences Department, College of Dentistry, King Saud University Riyadh, Riyadh, Saudi Arabia; b Oral Medicine and Diagnostic Sciences Department, College of Dentistry, King Saud University Riyadh, Riyadh, Saudi Arabia; c Oral Medicine and Diagnostic Sciences Department, College of Dentistry, King Saud University Riyadh, Riyadh, Saudi Arabia; d Oral Medicine and Diagnostic Sciences Department, College of Dentistry, King Saud University Riyadh, Riyadh, Saudi Arabia; e Faculty of Dentistry, Royal College of Surgeons, Ireland, Dublin.

**Keywords:** malignant lesions, multifocal oral leukoplakia, oral pathology, PVL

## Abstract

Proliferative verrucous leukoplakia (PVL) is a multifocal, slowly evolving lesion that resists all types of treatment and has a high propensity for malignant transformation into oral squamous cell carcinoma. Lack of awareness and acquaintance with white lesions of the oral cavity makes it difficult to diagnose. Besides being rare, PVL significantly aggressive, so clinicians need to be aware of it carefully. Therefore, it is recommended to have the earliest possible diagnosis and total excision of this lesion. We report this case to present typical clinical and histologic features of PVL so a For the purpose of sensitizing clinician.

**Patient concern:**

A 61-year-old female came to the clinic concerning of recurring painless, white patch on the tongue 2 months ago, associated with oropharyngeal dryness.

**Diagnoses:**

This case satisfies these major and minor criteria to diagnosed PVL.

**Intervention:**

An excisional biopsy of the lesion was done to check for the presence of dysplasia, as lesions were persisting. Hemostasis was achieved with single interrupted sutures.

**Outcome:**

no recurrence has been observed since excisional 1 year follow-up.

**Lesson:**

The key feature is early detection, precisely in cases of PVL it is critical for better treatment outcomes, lifesaving, quality-of-life enhancement. To detect and treat any potential pathologies, clinicians should meticulously examine the oral cavity and patients have to be aware and informed of the importance of regular screenings. This lesion is resistant to the presently available treatment modalities; therefore, total excision with free surgical margins is critical combined with a lifelong follow-up.

## 1. Introduction

Proliferative verrucous leukoplakia (PVL) was initially identified by Hansen et al^[1]^, in 1985 as a discrete variety of oral leukoplakia (OL). This presents with multifocal, slowly evolving lesion, resisting all types of treatment, with a high recurrence rate and a propensity for malignant transformation. This pathology tends to follow a persistent clinical course with high rates of resurgence and gradual development into oral squamous cell carcinoma.^[2,3]^ As this entity lacks distinct histopathologic criteria, the diagnosis is formed on the combined clinicopathologic evidence of progression.^[4,5]^ PVL is identified as primarily a clinicopathologic entity. PVL starts as a simple white patch or plaque, with marked hyperkeratosis of the mucosa and has a tendency for contagious spread. PVL is also typified by its progressive, relentless, and irreversible feature, and with time involved areas show an exophytic, wartlike growth or verruciform proliferation.^[1,3]^ The surface of the lesion shows aberrant keratinization initially and turns into extremely textured, varied, nodular, and indurated in due course.^[3]^ Heterogenous appearance and progressive clinical course is the hallmark of PVL.^[2]^ This lesion usually multifocal that frequently involves the buccal mucosa, gingiva, and edentulous alveolar ridge. It slowly spreads to contagious or noncontagious regions.^[6–9]^ A strong female predilection, with a 4:1 female-to-male ratio, is observed in PVL,^[10]^ and the average age at the time of diagnosis is around 70 years.^[9]^

The etiology of PVL is still unclear. This led to contemplation that it may be multifactorial.^[11]^ Tobacco-related products, chronic irritation, Human Papillomavirus, Epstein-Barr virus, Candida, certain oncogenes, genetic, and immunological influences are the possible contributing sources.^[1,2,12,13]^ Risk factors that are associated with OL and oral squamous cell carcinoma (various tobacco and arecanut preparations, betel leaf, alcohol) do not appear to be strongly linked with PVL.^[7]^ EBV, HPV, p53 expression, DNA ploidy, and expression of transforming growth factor-alpha have been investigated in PVL. None of these reports gave a clearer picture of their role in the etiopathogenesis of PVL.^[14]^ To date, no definite causal factors have been identified with PVL.^[15]^ It has been proposed that both external stimuli and internal elements are contributing factors along with immune dysfunction in precipitating PVL.^[16]^

A propensity to present as a multifocal lesion, which is resistant to most of the treatment and, high rate of recurrence (87–100%) and tendency to change into malignant lesion (60–100%), high mortality rates(30–50%) are indicative of PVL’s aggressive biologic behavior.^[7,17]^ The overall rate of malignant transformation ranges between 40% to100% in a follow-up period of 4.4 to 11.6 years.^[18]^ PVLs in the gingiva, palate, and tongue are frequently associated with malignant transformation.^[5,18]^ Frequent malignant transformation in PVL is indicative of it is capability for unrestrained progress.^[19]^ The proliferative influence of PVL was elucidated on the grounds of the high rate of field cancerization residing in PVL cases.^[17]^ Even the relentless, multicentric lesions resistant to interventions in PVL are also attributed to field cancerization.^[3]^ This exists as diffuse submicroscopic changes in oral mucosa associated with PVL.^[2]^

**Figure 1.**
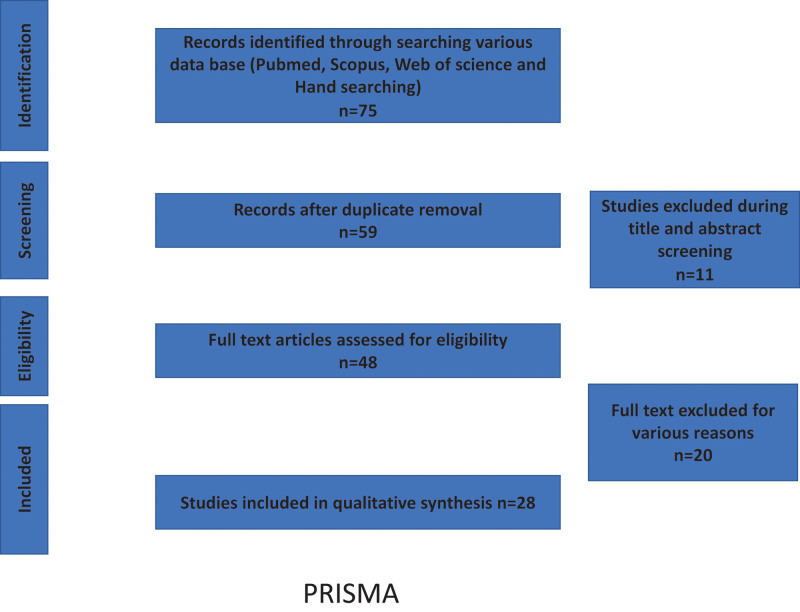
PRISMA.

The significance of the detection of this lesion lies in the experience of both the clinician and histopathologist that seemingly innocuous appearing verrucous pathologies, regardless of their color and despite being dysplastic, might in due course, progress into cancer.^[20]^ All PVL begins as homogenous OL, but not all clinical OL end in PVL. PVL is most likely to initiate patches of verrucous hyperplasia or carcinoma if left unattended.^[1]^ So early detection and treatment are essential. Most PVL cases reported are in elderly women with a long clinical course, exhibiting marked multifocal warty keratoses. Only a small number of early stage PVL cases are mentioned in the literature (Fig. 1). This report describes an unusual case of early PVL in a 61-year-old female patient with an immune system malfunction due to a long-standing autoimmune disorder and currently is in end-stage chronic kidney disease (CKD). Ethical approval obtained for this case report from King Saud University Institutional Review Board with reference No. 22/0827/IRB. “written informed consent” was obtained from the patients legal guardian.

## 2. Case report

A 61-year-old Egyptian female came to the Department of Oral medicine at King Saud University with a chief complaint of a painless, rough white patch in the mouth. She also had oropharyngeal dryness and teeth-related issues. Past medical history was significant. The patient has Sjogren syndrome, which was initially diagnosed as systemic lupus erythromatosus in 2013. Autoimmune sialadenitis features were noted on a minor salivary gland biopsy. Serology was supportive of this diagnosis then. Prednisolone, Plaquenil, and Pilocarpine were the medication taken for a few weeks to months. The patient is on Nifedipine for hypertension, and Thyroxin for hypothyroidism. At present patient’s serological and urine investigation report is suggestive of end-stage CKD, and patient undergoing dialysis every week. She has been advised for a renal transplant. Family history was noncontributory.

**Figure 2.**
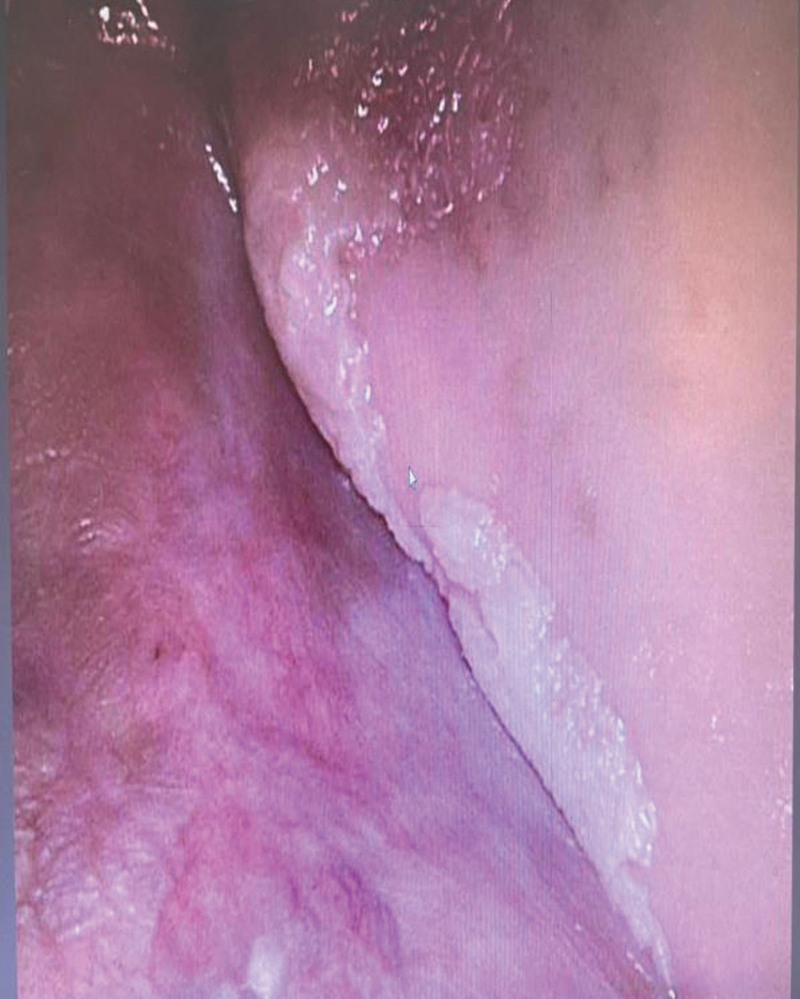
Lesion extending into the buccal vestibule, tuberosity, and palatally.

**Figure 3.**
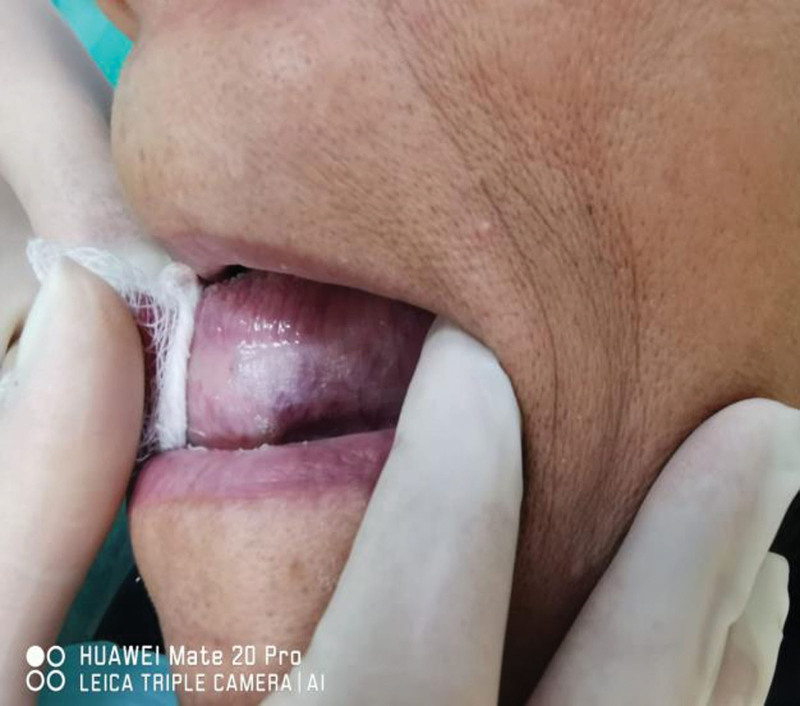
Lesion in the left lateral border of the tongue, extending anterior-posterior and ventrally.

**Figure 4.**
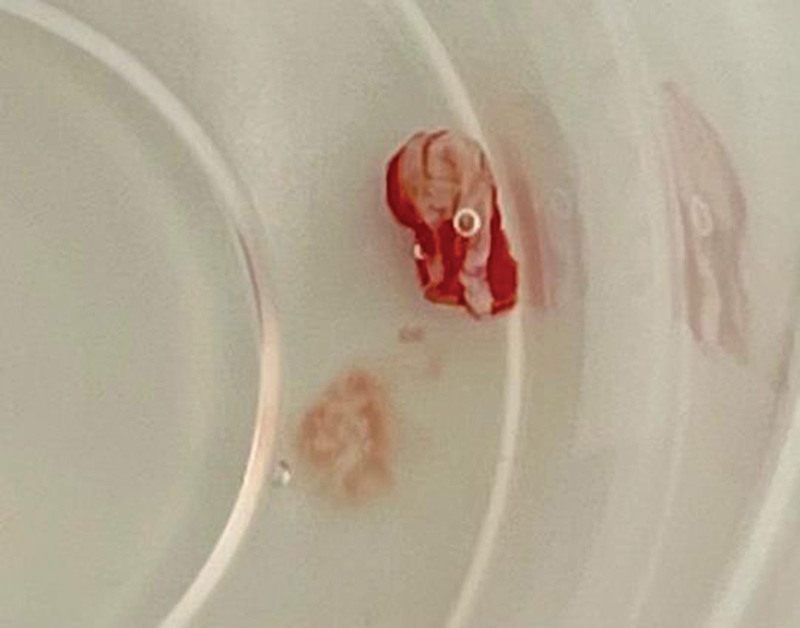
Specimen in 10% formalin solution.

**Figure 5.**
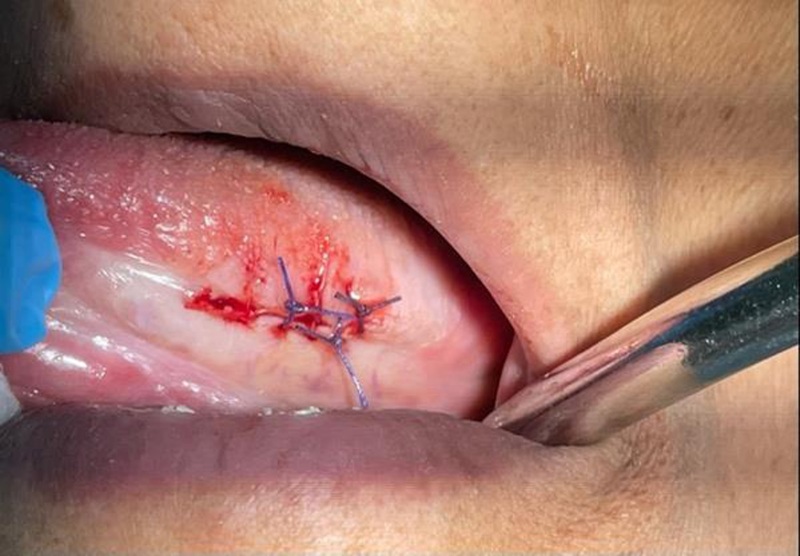
Hemostasis was achieved with single interrupted sutures.

General physical examination were showing features of CKD like swollen ankles and feet. She complains of tiredness, shortness of breath, difficulty sleeping and itchy skin. There was no palpable cervical lymphadenopathy on extraoral examination. On intraoral examination, multifocal white lesions were observed. A homogenous white plaque, with raised, corrugated surface and well-defined borders over the left maxillary alveolar ridge in the molar region was noted. The lesion was extending into the buccal vestibule, tuberosity, and palatally (Fig. 2). approximately 2 × 3 cm. The lesion was coarse, firm, non-scrapable, and non-tender on palpation. A contagious homogenous faint white patch over the left buccal mucosa with marginal melanotic hyperpigmentation was also noted. A white pseudomembranous coating suggestive of oral thrush was observed on the dorsal surface of the tongue. Another homogenous, non-scrapable, white patch, measuring approximately 2 × 3 cm in size noted in the left lateral border of the tongue, extending anterior-posterior and ventrally (Fig. 3). These multifocal lesions were asymptomatic. The patient did not have any adverse habits. There was no obvious source of irritation such as a sharp broken tooth or a fractured restoration. A provisional diagnosis of frictional keratosis was made. OL, chronic hyperplastic candidiasis, oral lichen planus (OLP), and PVL were considered differentials. The patient was kept under observation. Patient education and assurance were provided. Use of topical antifungal medication and chlorhexidine mouth wash was advised for 2 weeks. After achieving local anesthesia of mepivacaine 2% combined with epinephrine (1:100 000) around the biopsy site, an excisional biopsy of the left lateral border of the tongue lesion done to check for the presence of dysplasia, as lesions were persisting by long elliptical incision around the lesion with a size 15 blade rather than a round shape incision that won not close adequately done with deep cut down taking a couple of millimeters on each side of the lesion going well down into the muscular pad parallel to the blood vessels then tie off any bleeders vessel that is evident and close the muscle tissue and the overlying mucosa, lesion removed and placed at 10% formalin solution (Fig. 4). The procedure done without any active major bleeding only one small vessel that we dealt with by using a hemostat clamp grasp the vessel and Hemostasis was achieved with single interrupted sutures (Fig. 5).

**Figure 6.**
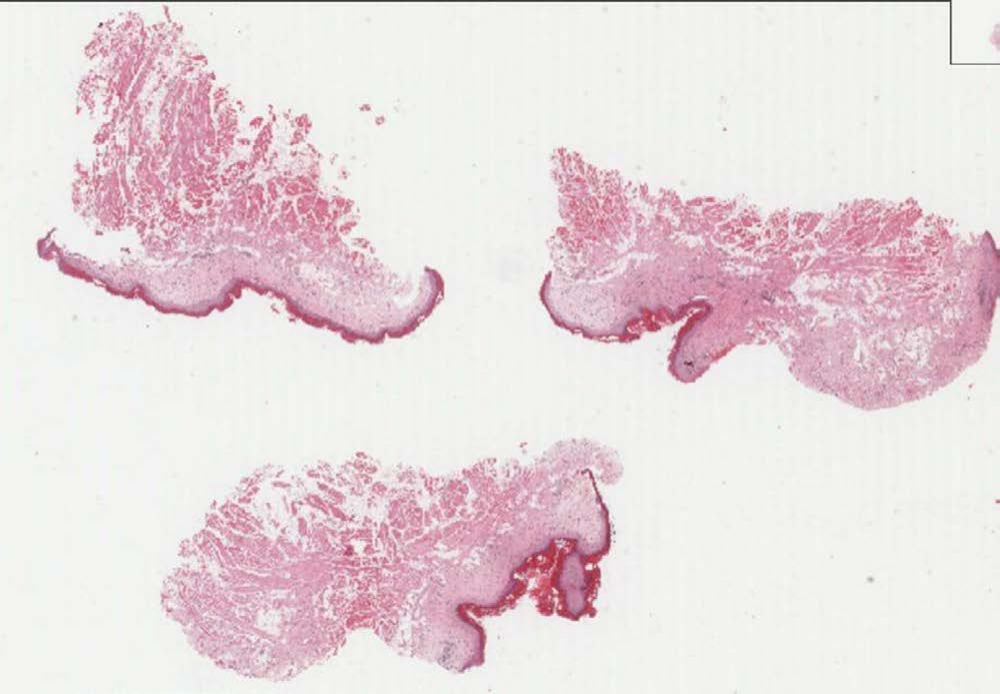
Photomicrograph shows stratified squamous epithelium in a wavy-like undulation with hyperkeratosis and focal interface mucositis. (Scanner View, H&E).

Microscopy revealed proliferative stratified squamous epithelium in a wavy-like undulation with marked hyperorthokeratosis and a prominent granulosum layer. There was a generalized loss of retepegs. Mild cytological dysplastic change was noted in the epithelium. Microscopy was suggestive of OL with mild dysplasia. A band-like inflammatory infiltrate noted in focal areas at the interface. Photomicrograph shows stratified squamous epithelium in a wavy-like undulation with hyperkeratosis and focal interface mucositis. (Fig 6 Scanner View, H&E). Focal basal cell degeneration, apoptotic cells, and melanin incontinentia, resembling lichenoid reaction were noted. Densely fibrous lamina propria with scattered inflammatory cell infiltrate noted. Submucosa consists of skeletal muscle, adipose tissue, and nerve fibers. This lesion demonstrated an undulating epithelium, corrugated hyperorthokeratosis with minimal cytological changes, which is indicative of architectural changes observed in epithelial dysplasia. White lesions involving the tongue, alveolar ridge, and buccal mucosa in this old woman depict a multifocal presentation along with an exophytic corrugated lesion on the ridge indicator of a distinctive form of OL, and evolving PVL or early PVL of the oral mucosa.

Excision of these oral lesions along with a thorough histopathologic examination to confirm the diagnosis has been advised, as PVL is a retrospective clinical diagnosis (Fig. 6). The patient is under close follow-up.

## 3. Discussion

PVL is a clinical diagnosis for OL that includes a range of clinicopathologic stages which is prone to show reoccurrence and, occasionally, the clinicopathologic characteristics of malignancy.^[2]^ “Proliferative multifocal leukoplakia” has also been suggested for this pathology. Based on the fact that multicentric presentation is a more salient criterion than a verrucous appearance and verrucoid presentation may not be evident in the initial stages.^[21,22]^ Even “proliferative leukoplakia” or “proliferative erythroleukoplakia” has been proposed as PVL lesions can be fissured (18%), erythematous (22%), and unifocal.^[9]^

Histopathologic findings related to PVL are dependent on site, the extent of the lesion, and satisfactory biopsy. Hansen et al^[1]^, observed that PVL lesions encompass a spectrum of histopathologic changes.^[18]^ It is microscopically graded on a scale from 0 to 10, denoting a continuum of severity as suggested by Hansen et al In the present case, the white lesion on the left lateral border of the tongue is Grade 2, hyperkeratosis (clinical leukoplakia).

Several authors have proposed a group of diagnostic criteria related to the diagnosis of PVL.^[5,23,24]^ As it lacks specific histological criteria, the diagnosis of PVL is based on combined clinicopathologic proof of progression.^[18]^ However, currently, there is no criterion that will allow for the early diagnosis of the disease.^[18]^ This case satisfies these major and minor criteria. A (multifocality; lesions involving the tongue, buccal mucosa, alveolar ridge) and E (histopathology; marked hyperorthokeratosis with mild dysplasia) criteria combined with minor b (gender; female) and c (smoking habit; nonsmoker) criteria.

In the present case, the biopsy section showed an intense inflammatory infiltrate in the superficial lamina propria with a lichenoid pattern (interface mucositis). It is noted that in the early stage of PVL, lesions generally reveal a junctional activity with infiltration of lymphocytes. This might have a distinct lichenoid pattern characterized by basal degeneration, with eosinophilic apoptotic bodies resembling various forms of oral lichenoid stomatitis like OLP. Hence, PVL has no single defining microscopic feature.^[2,17]^ The progressive microscopic findings of PVL might have features that can be confused with OLP. Lichenoid morphology might be the initial presentation, an early form of PVL.^[22]^ It has been proposed by several authors that the early stage of PVL can reveal a lichenoid feature in a biopsy, though the clinical appearance would be like an OL, as in the current scenario. PVL, OLP, and oral lichenoid lesions are dynamic pathologies that bring in various clinicopathologic findings during their progression.^[25]^

Several authors have expressed the difficulties in the early recognition and differentiation of PVL due to overlapping clinicopathologic features between PVL and conventional OL.^[15]^ Due to their shared findings, early detection of PVL is generally delayed. Epithelial dysplastic features are more frequently noted in the conventional OL than in PVL. PVL lesions might show some atypical cytologic features, but the architectural disarrangement is immensely dominating in PVL.

PVL is usually resistant to conventional management statergies.^[10]^ Photodynamic therapy has been advocated by several researchers for multiple focal lesions concurrently.^[10]^ To control PVLs unchecked growth and malignant transformation early recognition and aggressive treatment are recommended.^[19]^ Generally, aggressive surgical resection is the treatment option carried out.^[10]^ Multicentric presentation of PVL requires several biopsies to rule out malignancy and periodic long-term follow-up is mandatory.^[10]^ Recently the treatment goal has been shifted from cure to control as it is a practical approach.^[15]^

## 4. Conclusion

A proper diagnosis of white lesions of the oral cavity is perplexing for most clinicians. Lack of awareness and acquaintance with this entity makes it difficult to diagnose. A strong clinicopathologic judgement is needed for a correct diagnosis of PVL. Several sequential biopsies from different sites may be necessary due to it is multi-stage development. Persistent and progressive chronic proliferation, multiple occurrences, refractory to treatment options, high malignant transformation, and mortality rate are the salient features of this entity. This pathology requires an early diagnosis and aggressive treatment to better the possibility of a favorable outcome.

## Author contributions

**Data curation:** Lujain AlSahman, Roba AlSahman.

**Investigation:** Abdullah Alsoghier, Hamad AlBagieh, Rana Alshagroud.

**Methodology:** Lujain AlSahman, Roba AlSahman.

**Writing – original draft:** Lujain AlSahman, Roba AlSahman.

**Writing – review & editing:** Lujain AlSahman, Roba AlSahman.
